# Multi-scale habitat preference analyses for Azorean blue whales

**DOI:** 10.1371/journal.pone.0201786

**Published:** 2018-09-28

**Authors:** Laura González García, Graham J. Pierce, Emmanuelle Autret, Jesús M. Torres-Palenzuela

**Affiliations:** 1 Applied Physics Department, University of Vigo, Vigo, Spain; 2 Instituto de Investigaciones Marinas (CSIC), Vigo, Spain; 3 CESAM & Departamento de Biologia, Universidade de Aveiro, Aveiro, Portugal; 4 Laboratoire d’Océanographie Physique et Spatiale, IFREMER, Brest, France; Universidade de Aveiro, PORTUGAL

## Abstract

Blue whales are sighted every year around the Azores islands, which apparently provide an important seasonal foraging area. In this paper we aim to characterize habitat preferences and analyze the temporal distribution of blue whales around São Miguel Island. To do so, we applied Generalized Additive Models to an opportunistic cetacean occurrence dataset and remotely sensed environmental data on bathymetry, sea surface temperature, chlorophyll concentration and altimetry. We provide a brief description of the oceanography of the area, emphasizing its high spatio-temporal variability. In order to capture this dynamism, we used environmental data with two different spatial resolutions (low and high) and three different temporal resolutions (daily, weekly and monthly), thus accounting for both long-term oceanographic events such as the spring bloom, and shorter-term features such as eddies or fronts. Our results show that blue whales have a well-defined ecological niche around the Azores. They usually cross the archipelago from March to June and habitat suitability is highest in dynamic areas (with high Eddy Kinetic Energy) characterized by convergence or aggregation zones where productivity is enhanced. Multi-scale studies are useful to understand the ecological niche and habitat requirements of highly mobile species that can easily react to short-term changes in the environment.

## Introduction

The archipelago of the Azores is like an oasis in the middle of the Atlantic Ocean. It is located about 2000 km SE of Newfoundland and 1500 km west of continental Portugal. In spite of being within the boundaries of the oligotrophic North Atlantic Subtropical Gyre [1], these islands are a key habitat for pelagic organisms. This is due to the presence of oceanographic features like eddies and fronts and their interaction with the irregular topography, which enhances biological productivity at different time and spatial scales. This dynamism favours the development of bottom-up processes across trophic levels and drives biomass aggregation at convergence zones, thus creating suitable foraging hotspots [2–5].

Cetacean habitat is often identified in relation to certain water depths or sea bed slope e.g.[6,7] but habitat preferences are better described by also taking into account oceanographic characteristics such as productivity or temperature. Dynamic variables are frequently used as proxies for prey availability or abundance, notwithstanding the temporal lags and spatial displacement that may occur between physical processes and the biological responses [8].

Around the Azores, 28 different species of whales and dolphins have been reported [9]. Seven of these are baleen whales, including the blue whale *(B*. *musculus)*, which is listed by the IUCN as an endangered species since its population has decreased by at least 70%, and possibly 90% over the last three generations, assuming a 31-year average generation time [10]. There are four recognized subspecies, of which only one is found in our study area, the Northern blue whale *(B*. *m*. *musculus)* [10]. Blue whale populations have been extensively studied in the eastern Pacific [11,12], Antarctic [13–15] and in some Atlantic areas such as the Gulf of St. Lawrence [16–18] and the central and NE North Atlantic [19,20]. Some individuals undertake annual migrations between the low latitude breeding areas and the north Atlantic feeding grounds, while others are thought to be resident in high productivity habitats [10]. Although they are seen in spring in mid-Atlantic waters around the Azores, apparently while on their northward journey [21], studies about them in the region are still scarce. This is partly due to the high logistic complexity and economic cost of dedicated sea surveys, which usually constrains the data available spatially, temporally or to a small sample size, not only for blue whales but also for other cetaceans [22–24]. Therefore, the use of opportunistic data sources such as whaling, stranding records, observations from fishing vessels, or more recently, sightings from whale watching boats, becomes increasingly important to complement and improve the existing knowledge of cetaceans in the region [9,21,25,26].

Whale watching activities started in the main Azorean islands in the early 1990's, and offer a cost-effective method to obtain highly valuable data on cetacean distribution and behaviour. Such opportunistic data collection has been proven to be a useful source of information to better understand the occurrence and habitat preferences of cetaceans worldwide and to obtain data that are otherwise inaccessible, especially where there is a lack of baseline data [27–29]. Even with some limitations, such as poorly quantified sampling effort or logistical constraints imposed by the nature and primary purpose of the platform (tourism), there are clear advantages such as inexpensiveness and good spatial and temporal coverage on a regular basis [8,29], especially regarding rare species [30]. Hence, knowing the strengths and weaknesses of each dataset beforehand is a key point in order to maximise its use while avoiding false conclusions or misconceptions.

Previous research on baleen whales in the Azores examined the timing of their arrival in relation to the spring bloom and their foraging behaviour around the archipelago before they continue to travel further north [21,24]. Their distribution has been studied in relation to sea depth [9] and several oceanographic variables with monthly resolution [31,32]. So far, other temporal scales have not been adequately investigated, although some recent studies suggest that cetaceans, as highly mobile species, and particularly in very dynamic environments, may be affected by both short- and long-term ocean variability [33–35].

As a migratory species, blue whales may be attracted to the Azores by persistent oceanographic events (e.g. spring bloom) which are usually quite predictable and can be detected based on coarse (temporal and spatial) scale oceanographic data. Monthly composites of satellite variables can provide the necessary evidence, and can help to reduce data loss apparent at finer scales caused by the great abundance of clouds in the area. However, when investigating habitat preferences of highly mobile marine species such as blue whales, capture of finer-scale oceanographic processes turns out important, as the animals can easily react to short-term environmental changes in order to better exploit the available resources. Thus, finer scales are known to improve model performance [36,37], so consideration of weekly and/or daily values is highly recommendable.

Identifying areas of likely occurrence of blue whales and other cetaceans is a necessary first step to correctly identify Important Marine Mammal Areas (IMMA) [38] or Marine Protected Areas important for cetaceans [39,40] to achieve conservation and management goals. Furthermore, the Marine Strategy Framework Directive (MSFD) requires for every EU Member State to assess its current level of environmental monitoring in order to improve its conservation measures, which usually implies collection of data on the status of various ecosystem components, including cetaceans [41]. New trends towards dynamic ocean management are currently under discussion in order to better adjust conservation measures to the highly mobile nature of marine life and its rapidly changing environment [42,43].

In this paper we analyze temporal distribution and habitat preferences for blue whales off São Miguel (Azores). We use sightings collected between 2008 and 2014 from whale watching vessels and a set of bathymetric and oceanographic remotely sensed variables. We apply Generalized Additive Models (GAMs) to investigate relationships between whale presence and environmental conditions and thus to make inferences about their habitat preferences. GAMs are a non-parametric tool used to quantify linear or non-linear relationships between an independent variable and one or more explanatory variables [44,45]. GAMs have been widely employed to model cetacean distribution worldwide e.g.[46–51]. There have been few studies of blue whale habitat use in the Azores and these have used presence-only modelling techniques, such as Maximum Entropy (MaxEnt) [31,32]. In this study we modelled blue whale habitat use in the Azores at different temporal (monthly, weekly and daily) and spatial resolutions (low and high) in order to provide a deeper understanding of blue whale habitat ecology, getting the most from the different resolutions of the environmental data available.

## Methods

### Study area

**Fig 1:**
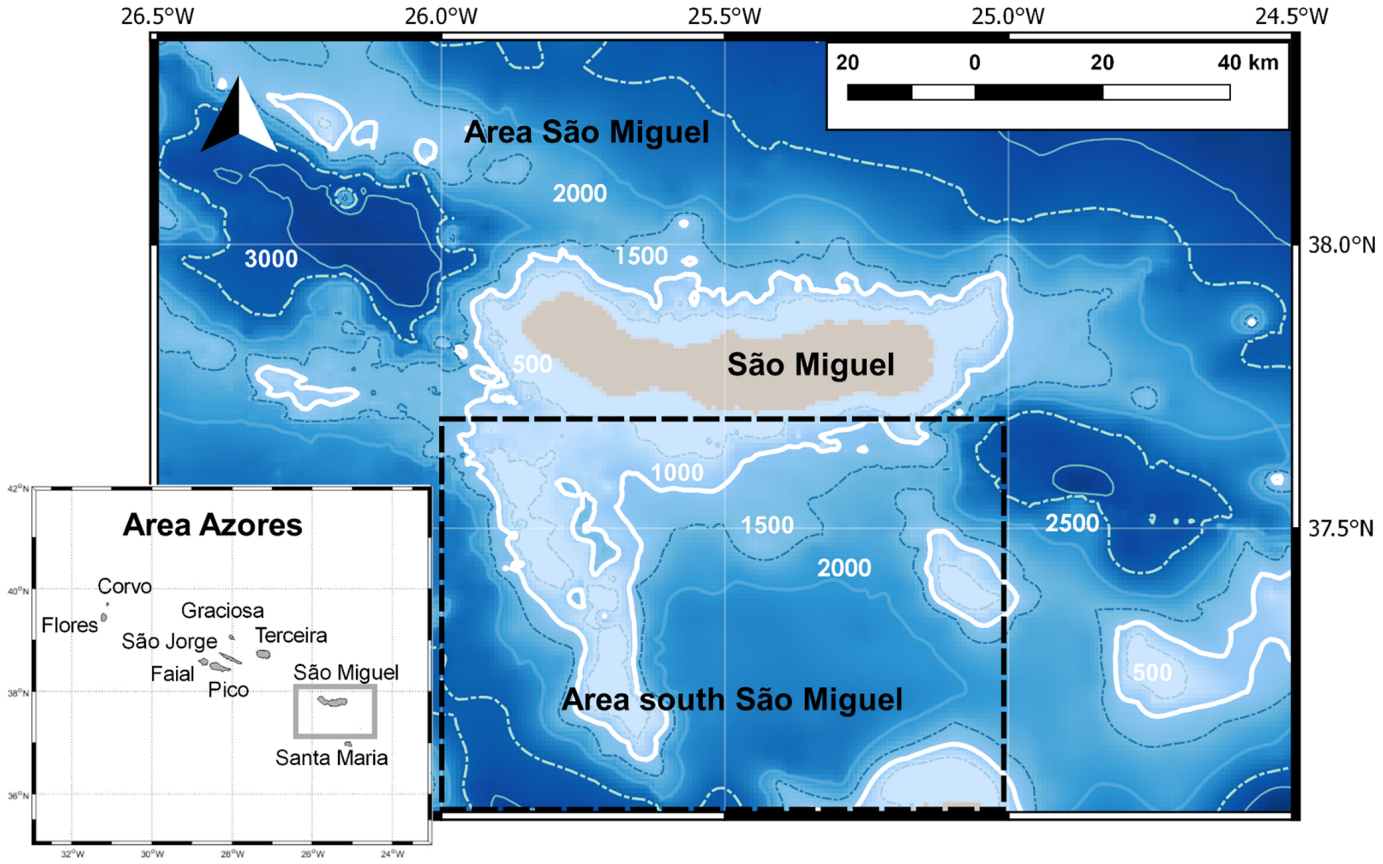
Azores archipelago with its nine islands and São Miguel area enlarged with bathymetry data. Depth contours each 500 m. Notice the three different areas considered for the environmental variables processing: area Azores (bottom left corner); Area São Miguel (grey square marked in Azores area and enlarged map); and area south São Miguel (dashed square).

The Azores archipelago is formed by nine volcanic islands located at 24–32°W and 36–41°N. They emerge abruptly from the deep mid-Atlantic waters (~4000 m deep), spreading along 630 km WNW-ESE and crossing the Mid-Atlantic Ridge. Our sea surveys were conducted off the south coast of São Miguel, the eastern-most island of the archipelago, located at 25–26.2°W and 37.3–38.1°N (Fig 1). Required licenses and permissions to operate the whale watching tours were provided by “Direção Regional dos Transportes” (Decreto Legislativo Regional 23/2007/A) and “Direção Regional de Turismo” (DLR 10/2003/A). The study area includes a great variety of marine habitats, from shallow waters over the very narrow continental shelf to deep waters, with depths of more than 2000 m relatively close to the shore. Sea surface temperature ranges from approximately 15°C in winter to 25°C in late summer. The Gulf Stream directly influences the archipelago with its cold branch, the North Atlantic Current (NAC), flowing south-eastward between 45 and 48°N. The Azores Front/Current System (AF/AC), characterized by strong salinity and temperature cross-gradients, passes south of the archipelago (between 32 and 37°N) forming the northern boundary of the North Atlantic subtropical gyre [52]. Both NAC and AF/AC are major sources of mesoscale oceanographic features [53] which favour the aggregation of marine life [5,8,54,55].

### Sightings

Cetacean sightings were recorded during the commercial trips of a whale watching company with base port in Ponta Delgada (São Miguel, Azores). Unlike many other opportunistic data sources, data were gathered throughout the year, in so far as the weather, the sea state and the number of tourists were good enough to conduct the trips. For each trip, experienced observers with binoculars (Steiner 20x80 mm), stationed at strategic points on land, typically started searching for cetaceans around one hour prior to launch and would continue observing and relaying this information to the boat, until cetaceans are sighted from the boat. Thus, cetaceans were located first from land and then boats were piloted directly towards the location of the animals, observing current legislation about approaching free-living cetaceans (Decreto Legislativo Regional n° 10/2003/A and DLR 13/2004/A). Once there, biologists on board registered the location (GPS) and time of the sighting, as well as species, group size and composition, behaviour and other observations. As research was not the main purpose of the activity, collection of standardized data, always registered manually, was restricted so as to interfere as little as possible with the main goal, tourism. Therefore, routes were not recorded, but starting and finishing times of each trip were recorded.

**Fig 2:**
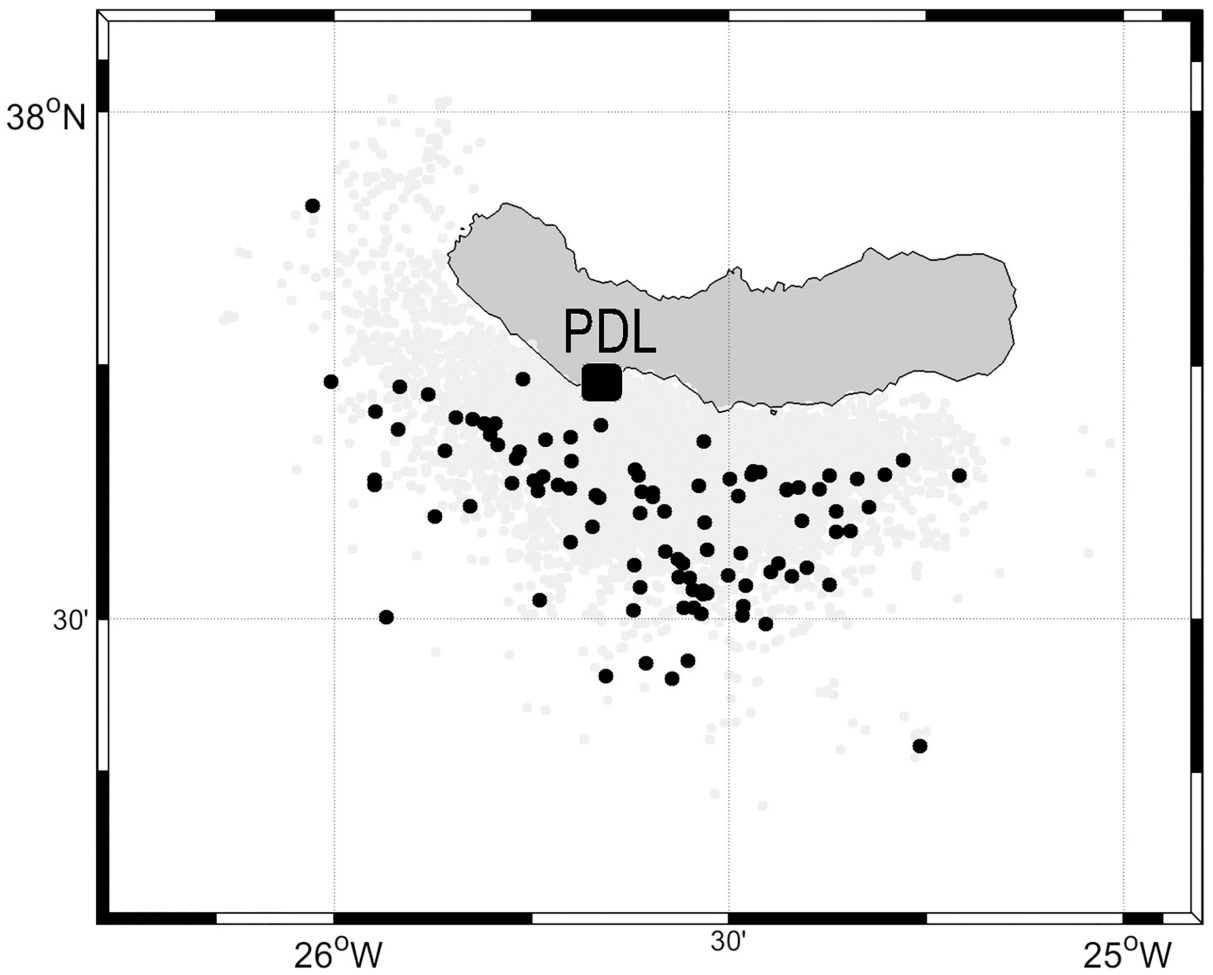
Map of the study area with all the sightings registered between May 2008 and December 2014. Black points are blue whale sightings (n = 89) and grey points correspond to the other species (n = 7711). Ponta Delgada is marked in the south coast as the base port.

Only those sightings for which the species identification was confirmed (genus accepted for beaked whales) and a reliable location recorded (longitude, latitude) were considered for the analyses (Fig 2). Following this process, around 93% of the sightings data were retained for analysis. The study period ran from May 2008 to December 2014.

### Effort

As animals were spotted firstly from land and sightings at sea are thus not associated with the amount of boat-based search effort during a trip, it is not possible to calculate real search effort or blue whale sightings per unit effort for this dataset. However, we recorded the number of trips, trip duration and time spent at sea each season and year as a rough indicator of the seasonal and year-to-year distribution of effort. As described below, we used sightings of other cetacean species as “absences” for blue whales.

### Environmental variables

The main environmental variables used for the analyses are summarized in Table 1. We obtained depth data from the General Bathymetric Chart of the Oceans (GEBCO, 2012) with a resolution of 30 arc-seconds. Slope was derived in degrees from the depth raster using the Surface Analysis tool from IDRISI Kilimanjaro. Each sighting was linked to its corresponding depth and slope values using Matlab R2012a. Distance to the coast from each sighting was calculated with ArcGIS 9.3, using as a reference high resolution coastline data provided by the *Instituto Hidrográfico de Portugal* in 2011.

**Table 1:** Main environmental variables from which the others were derived.

	VARIABLES	UNITS	SPATIAL RESOLUTION	TEMPORAL RESOLUTION	SOURCE
**PHYSIOGRAPHIC**	**Depth**	m	30 arc-seconds(~1 km)	static	GEBCO-08
	**Distance to the coast**	m	30 arc-seconds(~1 km)	-	Instituto Hidrográfico Portugal high resolution coast line
**OCEANOGRAPHIC**	**SST**	K (°C)	0.05° grid (~6 km)*(10–100 km effective)*	Daily	OSTIA
	**SST**	K (°C)	1 km	3–5 images/day	MetOp
	**CHL A**	mg/m^3^	1 km	Daily, 8-days	GlobColour
	**MSLA-UV**	m/s	0.25°	Daily	AVISO
	**WIND**	m/s	0.5° grid (~54 km)*(79 km effective)*	6 h	ECMWF

SST: Sea Surface Temperature. CHL A: chlorophyll a concentration. MSLA-UV: Mean Sea Level Geostrophic Velocity Anomalies. GEBCO-08: General Bathymetric Chart of the Oceans. OSTIA: Operational SST and Sea Ice Analysis. MetOp: Advanced Very High Resolution Radiometer (AVHRR) on board the Meteorological Operational satellite. GlobColour: European Node for Global Ocean Colour. AVISO: satellite altimetry data. ECMWF: European Centre for Medium-Range Weather Forecasts.

The distribution of a given species usually depends on the distribution of its prey. Oceanographic variables such as temperature or chlorophyll can act as proxies for primary production and prey availability or distribution. The oceanographic dynamic variables considered were prepared at three different temporal scales: daily, weekly and monthly. The products were chosen according to the availability of homogeneous data over the studied period. All the dynamic variables were processed with Matlab R2012a.

**Fig 3:**
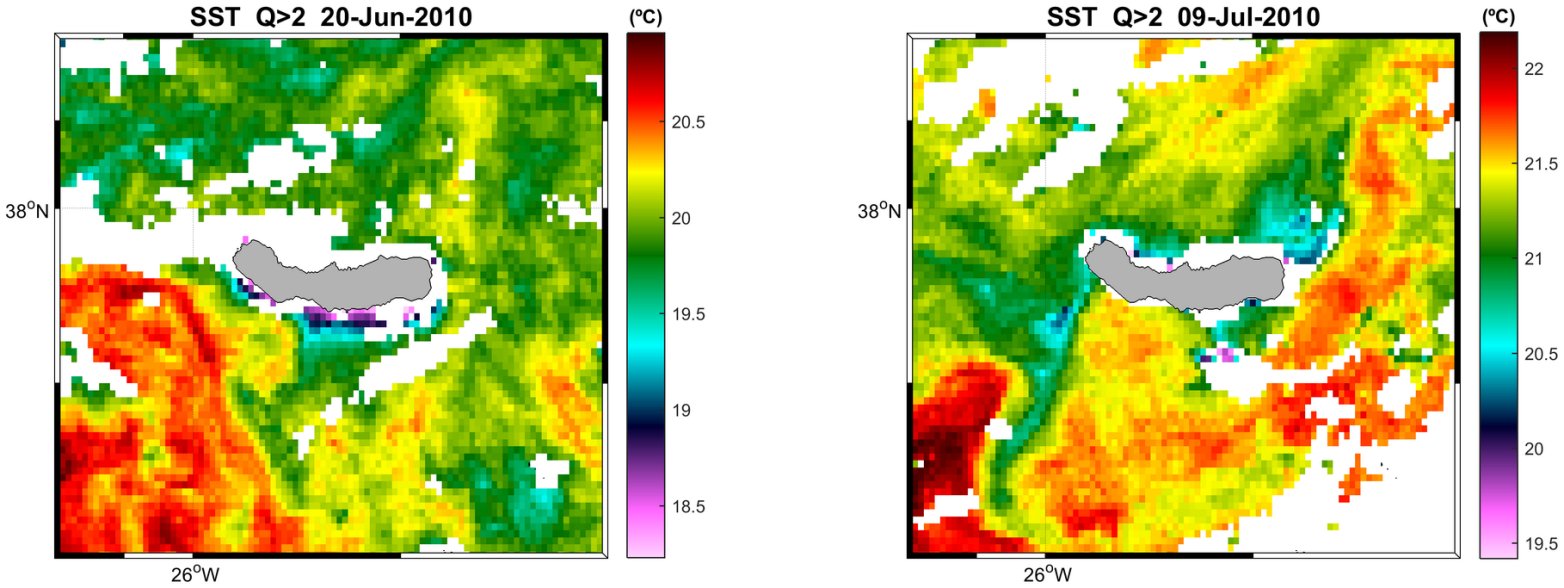
High resolution sea surface temperature daily map from MetOp data (only values with quality Index >2 were considered). Colorbar for temperature (°C). Blank spaces are missing values or bad quality values masked. Sharp gradients can be seen in the area, some with more than 2°C of difference in less than 20 km. **A)** Image from the 20^th^ of June 2010. It is clearly visible the cold water patch on the south coast of the island and a tongue of warm water retained by the platform on the south west. **B)** Image from the 9^th^ of July 2010. Cold water in the west and north side of the island, retained as well in the SW due to the interaction with the bathymetry.

Data on Sea Surface Temperature (SST) were gathered from two different products, one with high spatial resolution to get a detailed view of the study area and better account for the local oceanographic features (Fig 3), and one with coarser resolution that simplifies data processing and reduces data loss. The latter was derived from Operational SST and Sea Ice Analysis (OSTIA), which is run at the UK Meteorological Office on a daily basis. It combines high resolution products from infrared and microwave satellite instruments and *in situ* SST data, resulting in a foundation SST which avoids diurnal variability. Although interpolated onto a high spatial resolution grid (0.05°), this SST product exhibits a spatial resolution close to 50 km [56]. The high resolution product comes from the Advanced Very High Resolution Radiometer (AVHRR) on board the Meteorological Operational satellite (MetOp), which provides several daily images of SST with an effective resolution of 1 km (EUMETSAT/OSI-SAF, 2008). Each MetOp SST value has a quality index assigned, allowing us to exclude, for the purposes of the present analysis, the “unprocessed”, “not useable” and “bad” data. For both products, we extracted a value for each sighting location and day and also an average and standard deviation for São Miguel (37–38.5°N, 26.5–24.5°W) and for the Azores archipelago (35–42°N, 33–23°W). Weekly values were calculated as a 7 day moving average (± 3 days from the sighting date). A climatological mean (daily, weekly and monthly mean) was also calculated over the area for the 7 years of study to account for seasonality. A SST anomaly was calculated as the difference between the SST at the sighting location and the corresponding climatological mean for each observation. This allows us to account for local spatial or temporal variations. Furthermore, location of thermal fronts were calculated from SST images (data gaps interpolated) using a Canny edge detection function [57] with the upper threshold set at 1e-5°C/m (~1°C/100 km). Distance, gradient and SST of the nearest front point were calculated for every sighting.

**Fig 4:**
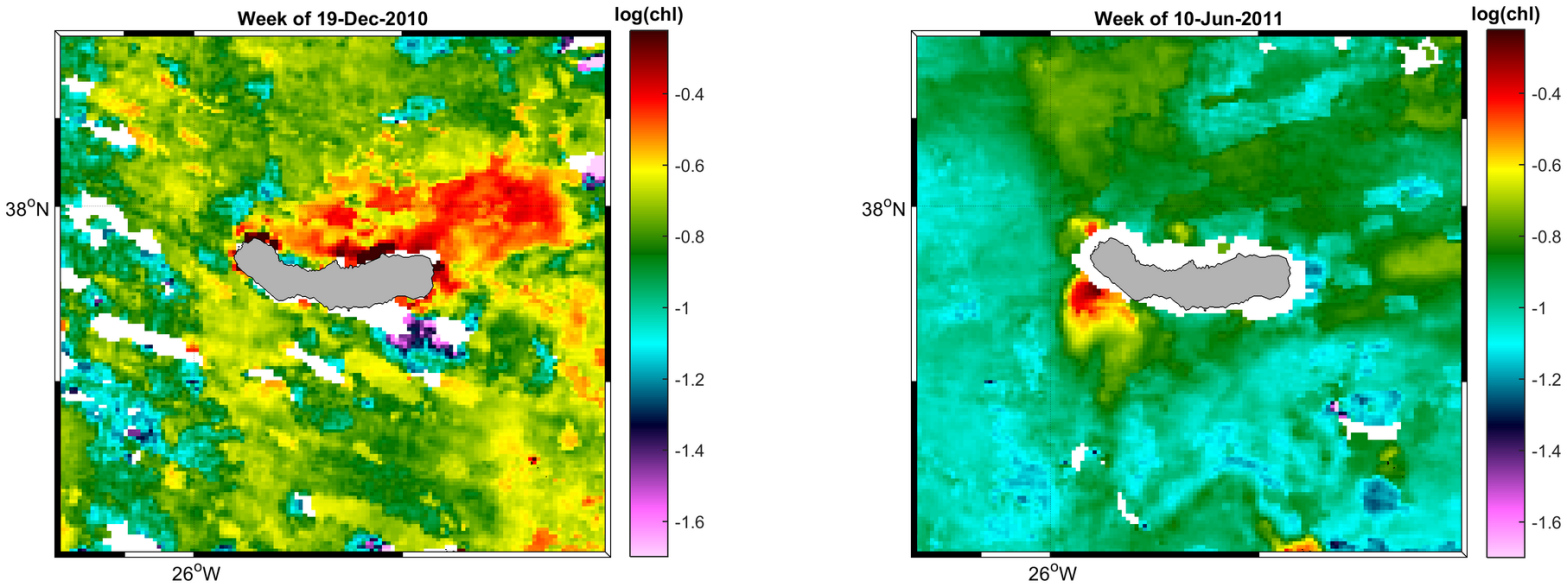
GlobColour images for chlorophyll a concentration. Warm colours represent higher concentrations of chlorophyll a. Blank spaces are missing values. Colorbar with log(chl a). **A)** Image from the 19^th^ of December 2010. Chlorophyll concentrates on the north side of the island, especially N of João Bom (NW) and off Praia da Viola (NNE). Chlorophyll concentration seems to expand further NE. **B)** Image from the 10^th^ of June of 2011. In this case, a local upwelling seems to occur in the W and SW of the island. It reaches quite high concentrations, but in a limited area.

Chlorophyll-a concentration is considered as a proxy for primary production. As blue whales feed mainly on krill, primary production should directly affect their prey availability with a relatively short time-lag. Krill development varies with environmental conditions, but juvenile stages can be reached in less than three (or even two) months after spawning, which usually happens after the phytoplankton bloom [58]. Chlorophyll data were obtained from GlobColour (http://globcolour.info) L3 products that combine, when available, data from SeaWIFS, MODIS, MERIS and VIIRS under a weighted average merging method, with weightings based on the sensor/product characterisation. As chlorophyll concentration is a dynamic variable, and its distribution can change in a short period of time, we decided to use daily, 8-day and monthly (processed from the 8-day ones) composites with 1km resolution. For each temporal resolution, we extracted a value for the sighting location, and a mean value and standard deviation over the Azores archipelago (35–42°N, 33–23°W), São Miguel (37–38.5°N, 26.5–24.5°W) and south of São Miguel (37–37.7°N, 26–25°W) (Fig 1). The latter was done in order to capture the effects of local upwelling, which has been detected on one side or the other of the island, based on the daily maps (Fig 4). The chlorophyll concentration of previous weeks (up to 17 weeks) and months (up to 4), i.e. before the sighting, was calculated to take into account the possible time lag between the phytoplankton bloom and the development of krill. Furthermore, three different chlorophyll indices were calculated as a ratio between the highest values of chlorophyll in a small area and the average chlorophyll concentration over a bigger area: chlorophyll Index 1 as the ratio between coastal São Miguel (37.65–37.75°N, 25.8–25.3°W) and São Miguel area (37–38.5°N, 26.5–24.5°W); chlorophyll Index 2 as the ratio between the south of São Miguel (37–38°N, 26–25°W) and a bigger area south of the archipelago (30–38°N, 32–22°W); and chlorophyll Index 3 as the ratio between the Azores area (35–42°N, 33–25°W) and a bigger Atlantic area surrounding the archipelago (30–48°N, 38–15°W). As blue whales are migratory, the different concentrations of chlorophyll close to the island compared with oceanic waters can help us to understand when or why the whales approach the studied area.

To account for the mesoscale variability of ocean dynamics in the area we calculated the Eddy Kinetic Energy (EKE) from altimetry data [55]. Gridded geostrophic velocity anomalies (Mean Sea Level Anomalies: MSLA-UV) computed with respect to a 20-year mean were produced by Ssalto/Duacs and distributed by AVISO (http://www.aviso.altimetry.fr/duacs/, accessed online on 6/07/2016, and now accessible at http://marine.copernicus.eu/). The data used were “Delayed Time” “all satellite merged” global daily maps on a 1/4° grid. The “All satellite merged” option was chosen because, during the entire study period, at least 3 satellites were available at any moment and, thus, the quality of the retrieved velocity field is enhanced with respect to the reference configuration merging [59], which uses observations from only 2 satellites. Horizontal and vertical components of the geostrophic velocity anomalies were used to calculate the EKE, which is defined as: 1/2((u-u_mean_)^2^+(v-v_mean_)^2^), where u_mean_ and v_mean_ are the temporal means of each velocity component over the whole study period [53,55]. In this case we consider monthly and seasonal means over the 7-year period, defining winter as December-January-February, spring as March-April-May, summer as June-July-August and autumn as September-October-November.

Wind data were obtained from the European Centre for Medium-Range Weather Forecasts (ECMWF) ERA-Interim 0’5 6-hourly surface analysis, which combines an assimilation system, constrained by the available *in situ* observations, and atmospheric modelling re-analysis. Its gridded resolution is 0.5° (~54 km) but the effective resolution is around 79 km [60]. Wind data were obtained only for the day and location of the sighting, in order to avoid misleading temporal and spatial averages.

### Variable selection

After preparing all the variables for the different spatial and temporal scales we had six main datasets: low spatial resolution monthly (47 variables), weekly (57) and daily (59), and high spatial resolution monthly (41 variables), weekly (60) and daily (60).

A detailed data exploration was carried out to assess collinearity among explanatory variables. We computed Pearson correlation matrices for each dataset in order to investigate the linear dependence between the predictors. These were visualized as dendrograms to better illustrate the similarity among the variables. The bigger the vertical distance between two variables, the more independent they are. We retained all the variables with a distance bigger than 0.8 from other variables [48,61]. The variables with distances under this value were selected firstly according to their horizontal distribution, retaining at least one of each cluster of correlated variables. When two highly collinear variables had similar ecological meanings, the one with most missing values was removed. For daily datasets, chlorophyll mean and standard deviation values from south of São Miguel were kept instead of those from around São Miguel to better account for local events. After this first selection, collinearity was assessed again based on the Variance Inflation Factor (VIF), removing all the variables whose VIF was higher than 5, one by one, starting with the highest value and recalculating VIFs at each step [62]. Finally, some additional variables were excluded from the high resolution selections (according to the dendrograms) due to greatly reducing (by hundreds and even thousands) the sample size available. After this process our datasets comprised of 19 (low spatial resolution daily), 18 (low spatial resolution weekly), 17 (low spatial resolution monthly), 17 (high spatial resolution daily), 19 (high spatial resolution weekly) and 14 (high spatial resolution monthly) explanatory variables.

### Temporal distribution

To assess the temporal distribution of whale sightings, taking into account the survey effort, we calculated an Encounter Rate (ER) as the number of sightings of blue whales divided by the total number of trips over each month or season. There were normally two trips per day (morning and afternoon). We consider the season as the period that included 99% of the blue whale sightings, hence March to June every year. We did not use data from 2008 in seasonal averages since data onboard were recorded only in surveys conducted during six months in 2008: from May to September and December.

### Habitat preferences analysis

Descriptive analyses were carried out in order to visualize available habitat conditions within the study area and understand distribution of the blue whale sightings in relation to habitat variables. The available area is considered as the Minimum Convex Polygon containing 100% of the sightings calculated with the “sp” and “adehabitatHR” packages for R [63,64,65]. It accounts for a total extension of 5780 km^2^. We determined the range of available values for the static variables in the estimated available area, although it should be noted that the true surveyed area is probably slightly larger.

To model species distribution, available environmental conditions need to be represented in the analysis [66]. Usually these “background” data are chosen at random to represent the range of environmental conditions in the study area, but this process can fail to account for sample bias, as sightings are generally recorded in more accessible areas [67]. Furthermore, the changing marine environment can present different conditions in the exactly same location at different times, therefore creating a different habitat. We decided to use a pseudo-absence approach in order to have effort-related ‘non-occurrence points’ and to reduce possible spatial bias [66,68]. To do so, we use the presence of the other cetaceans recorded during the study period as pseudo-absence points for blue whales [69], making sure to exclude occasions when blue whales were recorded at the same time as the other species. We thus ensured that those locations were sampled and that blue whales were not seen there at the given time, while accounting for, or at least reducing, sampling bias. The approach used here could be considered conceptually closer to the background sampling approach, as the number of “pseudo-absences” is, in this study, much bigger than the number of presences and the pseudo-absence dataset well characterizes the environmental conditions present across the study region [66]. Nevertheless, environmental conditions that were unsuitable for launching the boat (more prevalent in winter) would not have been included in the dataset or, therefore, our models.

Statistical analyses were carried out with the open-source software R 2.15.0 (R Development Core Team, 2012) and the mgcv library [70]. We applied Generalized Additive Models with a binomial distribution and a logit link function to each of the six datasets previously prepared in order to investigate habitat preferences of blue whales. The number of splines (*k* -knots which measure the complexity of the fitted curve-) was set to 4 for all explanatory variables to prevent overfitting and avoid unrealistically complex relationships. We used a backwards stepwise procedure to select the best model for each dataset, with removal of variables at each step being based on individual significance of explanatory variables and on the overall goodness of fit as measured by the Akaike Information Criterion (AIC) value. Starting from the first model with all possible variables, the least significant one (normally with highest p-value) was dropped out each time. If the resulting model had a lower AIC or a bigger sample size (and in the latter case was not a markedly poorer model according to the AIC value), it was retained. In some cases confidence bands were rather wide, even in the model with the lowest AIC, so extra model selections steps were needed [71]. In order to avoid a number of predictors higher than m/10, where m is the number of observations of the least represented category (here m = 89) [72], we retained in the final model only the variables significant at p<0.01. The best model for each dataset was achieved and the deviance explained was noted.

We applied a temporal k-fold cross-validation for each of the resulting models [73], using sightings from six of the seven available years as the training dataset, and the remaining one for evaluation (all but 2008 because no blue whales were recorded that year during the six months of data available). Then we compared the performance of each final model based on the Area Under the Curve (AUC) of the Receiving Operating Characteristic plot computed with pROC package for R [74]. AUC indicates how well the model adjusts to our presence/absence distribution without accounting for overfitting, so that more complex models usually have better AUC [75]. Hence, as described above, we attempted to avoid very complex models to carry out validation. We obtained an AUC for each year (i.e. for each option for the testing dataset) and an average AUC value and its corresponding standard deviation for each model, allowing us to compare the different models. We consider that a random model has a 50% chance to correctly distinguish between presence and non-presence locations, so we assume that any model with AUC higher than 0.5 should be better than random.

## Results

### Sea surveys

In total, 7711 sightings of twenty different species of cetaceans were recorded during the 2364 trips completed over 1386 non-consecutive days between May 2008 and December 2014 (Fig 2). Both resident and migratory species were sighted. Out of these records, 89 (1.2%) were blue whales *(B*. *musculus)*, registered during 85 trips over 70 days across the study period. There were 124 individuals recorded, mostly observed alone (71.9% of times) and with a maximum of three blue whales together in the same sighting. In three of the sightings, blue whales were observed together with common dolphins *(Delphinus delphis)*, while on six occasions they were observed together with fin whales *(Balaenoptera physalus)*, once reaching a total of at least ten whales in the same group.

### Effort

**Fig 5:**
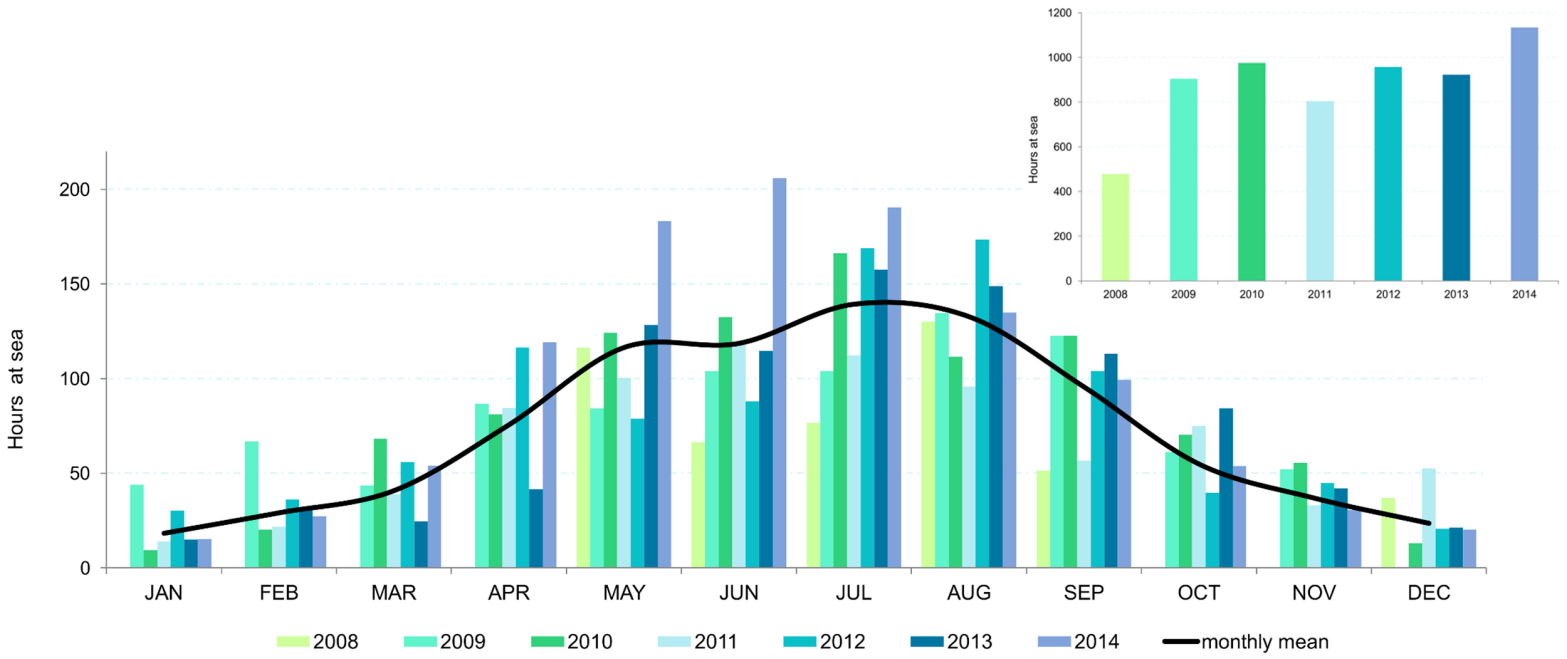
Time (hours) spent at sea per year and month. Rough approximation of the effort carried out over the seven years of study.

The average number of trips per year (excluding 2008, when there were only 6 months of sightings) is 338, although in both 2009 and 2014 there were more than 400 trips, and in 2011 there were only 269. Accordingly, the average time recorded at sea each year (excluding 2008) is 949 h, being highest in 2014 with 1134 h, and lowest in 2011 with 804 h (Fig 5).

The period of maximum effort corresponds to summer (June, July and August) when the 45.5% of the trips of the year were realized. The blue whale season, i.e. March to June every year, accounted for a total of 881 trips and 2462 h at sea. The number of trips recorded per year in this period ranged from 117 to 159, only higher in 2014, when 210 trips were carried out (mean 146.8 ±34.4). On average, 380 h (±96 h) were spent at sea per blue whale season, although in 2014 there were 563 h. Thus, effort coverage is reasonably consistent throughout the study period; and variation between months and years affects presences and pseudo-absences equally.

### Environmental variables

**Fig 6:**
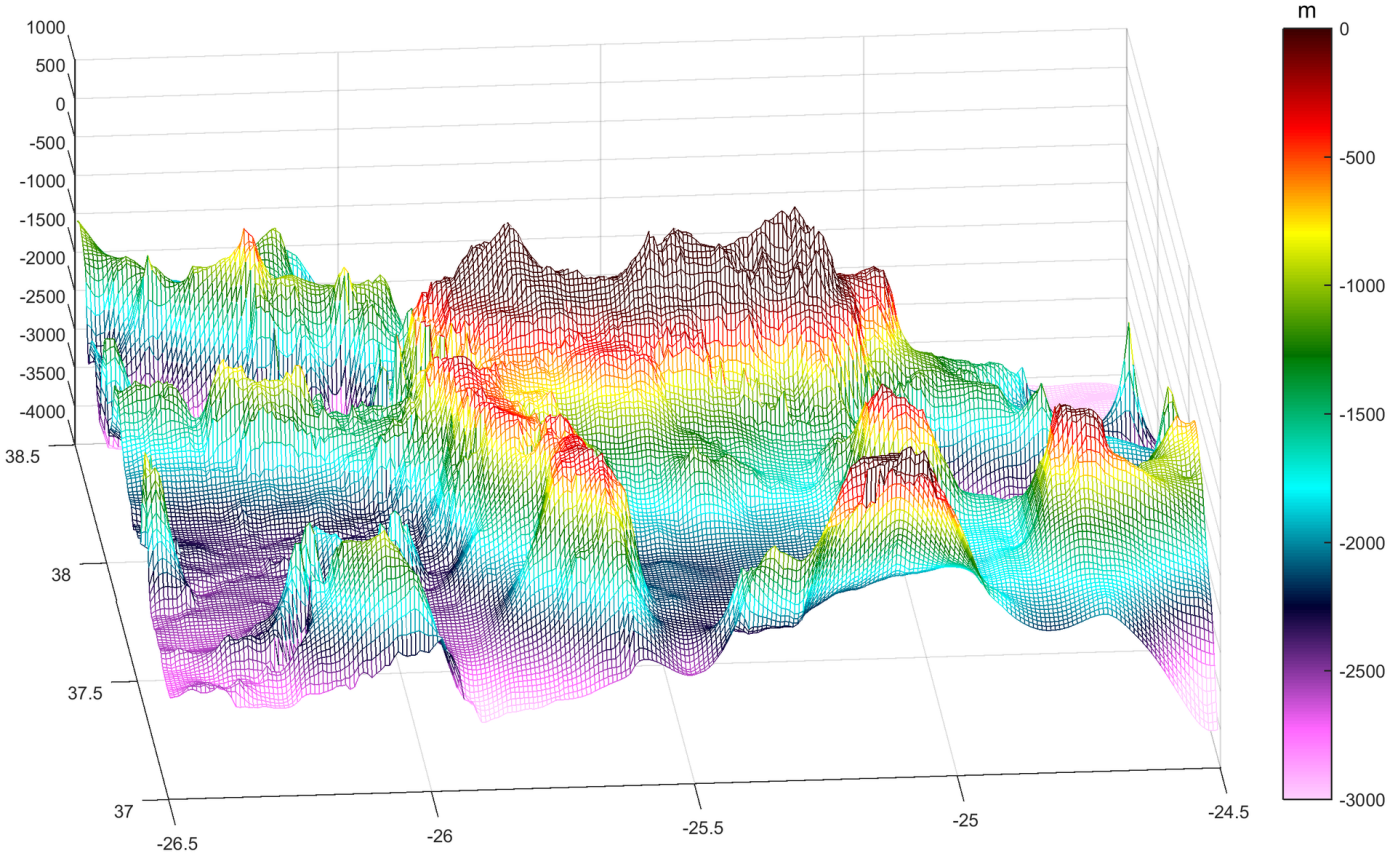
3D bathymetric map of the oriental group of the Azores. We can appreciate a more confined area in the south of São Miguel, enclosed by the shallower platform on the west, and submarine mounts and Santa Maria to the south. Latitude and longitude (degrees) represented on the x and y axis, and depth (m) in the z. The colour bar represents depth, going from dark blue as maximum depth and increasing up to red-brownish which represents land (above 0m).

Bathymetry data are consistent with the volcanic nature of these islands that abruptly emerge from the bottom surfacing almost without any surrounding continent shelf and resulting in the presence of very deep waters very close to the shore (Figs 1 and 6). Two deep oceanic trenches both reach depths of more than 3200 m at the NW (Fossa do Hirondelle) and SE (Bacia de São Miguel) of the study area. At the SW tip of the island, a shallower platform extends 60 km further south with an average depth of 500 m and a surrounding bottom of 2000 m. Considering the minimum convex polygon containing 100% of the sightings as the sampled area, the available sampled depths range from 0 up to 3237 m. Most of the sampled area (~60%) falls between 1500 and 2500 m depth, with ~13% between 2100 and 2300 m. The slope of the bottom ranges from 0 to 46.4°. 80% of the sampled area has a slope of <10°, while the highest slope values are found adjacent to the island and the submarine mounts and banks, and the smallest slope values (almost no slope) occur where the deepest waters are found.

Every year sea surface temperature reaches its minimum value in winter; minima during the study period range from 15.2°C in 2013 to 16.75°C in 2012. SST starts to progressively increase in April, and achieves its highest values (up to 24.7°C) in late summer (end of August, beginning of September). During the study period we noticed clear inter-annual differences. 2008 was warmer than the average, especially in summer. Winter 2012 was warmest, almost 2°C higher than the coldest year, 2010. In winter and spring of 2010, 2011 and 2014, the water was colder than in other years. Around the studied area it is not uncommon to find a surface temperature difference of 2°C between coastal and offshore waters (40 km from land), as well as sharp changes over short periods of time, as in September 2014, when SST dropped more than 2°C in less than two weeks.

Chlorophyll concentration is not particularly high in the Azores. Daily means around São Miguel ranged from 0.029 mg/m^3^ in late September 2011 up to 2.007 mg/m^3^ in April 2010. The concentration was usually below 1mg/m^3^, especially in summer when it rarely exceeded 0.2 mg/m^3^. In late autumn it starts to increase, achieving maximum values every year in spring, mainly in April, when the well known “spring bloom” takes place [24].

Looking at high resolution images for sea surface temperature and chlorophyll, although there were many cells with missing data due to the abundance of clouds, we detected local features on one side or other of the island, i.e. colder waters (Fig 3) or higher concentrations of chlorophyll close to the islands (Fig 4).

Eddy Kinetic Energy becomes higher in late spring-summer, with the Azores Front/Current System south of the archipelago (32–34°N) clearly visible in the seasonal maps. Winds around the archipelago are highly variable in strength and direction, but westerly winds seem to be generally strongest, which was particularly noticeable in winter 2009 and winter-spring 2010.

### Temporal distribution of blue whales

**Fig 7:**
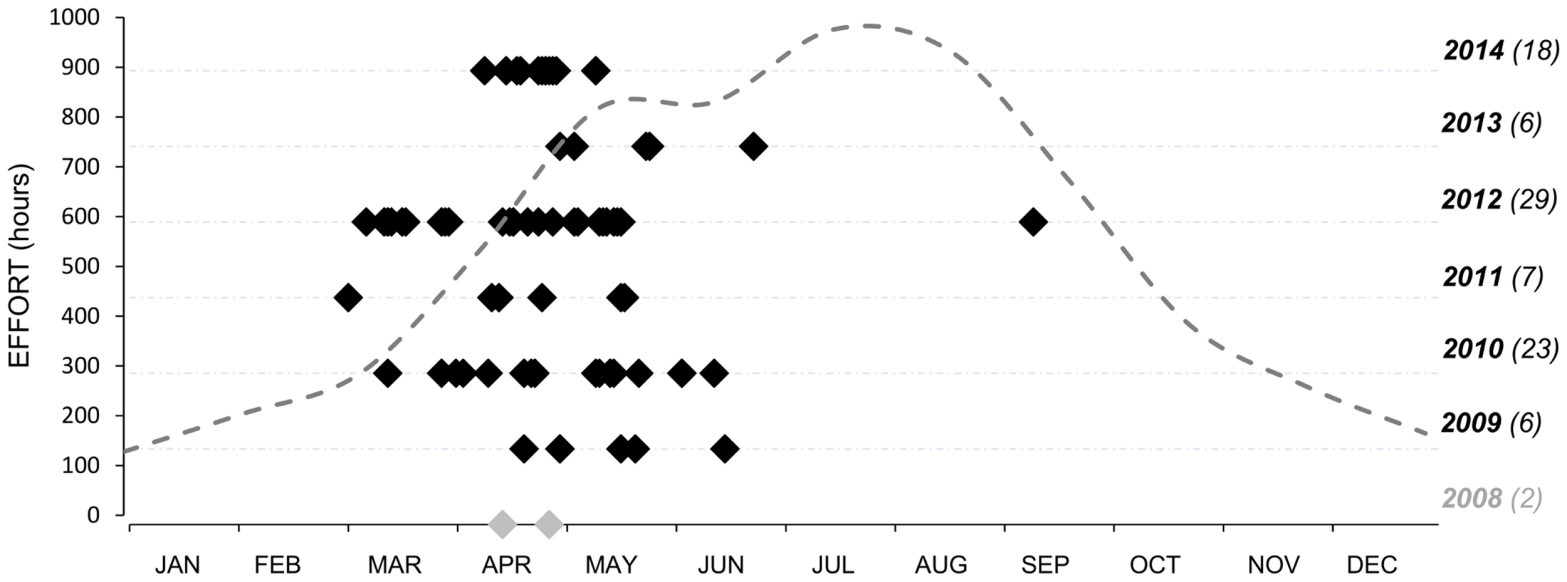
Temporal distribution of blue whales between May 2008 and December 2014. Dashed line shows the effort recorded, thus, hours spent at sea (left axis) each month along the 7 years of study. On the right of the graph, the year with the corresponding number of blue whale sightings in brackets is indicated. Sightings made in 2008 are grey, due to the late beginning of the data collection.

Blue whales were sighted regularly every year (except in 2008) between March and June, with only one sighting outside this period (a whale recorded in September 2012) (Fig 7). The overall seasonal ER (excluding 2008) was 0.10 sightings per trip, but ER was particularly high in 2010 (0.16) and 2012 (0.23), the latter being the year in which the highest number of blue whale sightings was recorded (n = 29). The highest monthly ER was in April (0.19), when 42.9% of the sightings were registered. April 2014 was by far the month with most blue whale sightings (18% of the total) within the study period. Inter-annual variation was also found in the number of sightings of blue whales recorded, with most sightings (29) in 2012.

### Habitat preferences

#### Descriptive analyses

**Fig 8:**
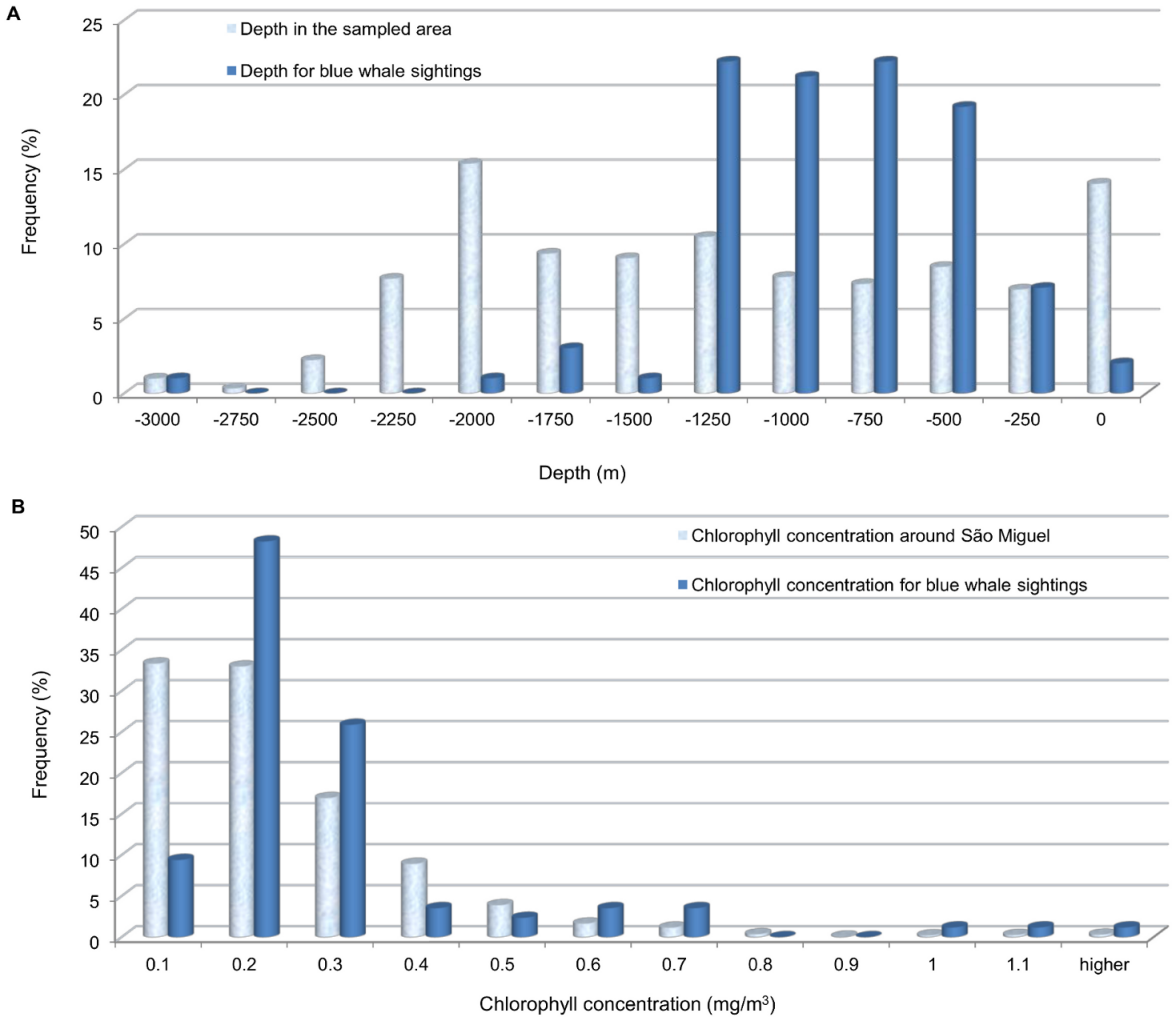
Histograms of environmental values recorded in the study area against the ones associated with blue whale sightings. A) Depth. B) Daily chlorophyll around São Miguel. It is noticeable how blue whales occur across a much smaller range of conditions than the available in the habitat.

Blue whales were sighted in waters of a wide range of depths (126–3000 m), but 83.2% of them were in waters between 500 and 1500 m depth (median: 987.6m), although this depth occurred in only 33% of the sampled area (Fig 8a). They were mostly (86.5%) sighted over slopes of less than 7.5° (range: 1–20.55°, median: 3.99°). Regarding sea surface temperature at the sighting location, the whales were mostly seen in cold waters (75.3% of them between 15 and 17°C), although they were sighted in waters with SST ranging from 14.4° up to 22.1°C (median: 16.31°C). Despite the general low chlorophyll concentrations in the study area (66.3% of the daily values are below 0.2 mg/m^3^), most of the blue whales (90.6%) were found when concentrations were relatively high (>0.1 mg/m3) around São Miguel (Fig 8b).

#### GAM results

The three low spatial resolution models, daily, weekly and monthly, performed reasonably well in terms of deviance explained (33.7%, 38.7% and 40.7% respectively) and AUC (0.913, 0.918 and 0.936). They all show a small between-year standard deviation in AUC (0.031, 0.036 and 0.025), thus fitting reasonably well for all the studied years (Table 2).

**Table 2:** Summary of the GAM results for the six obtained models.

		LOW SPATIAL RESOLUTION	HIGH SPATIAL RESOLUTION
**DAILY**	**AUC**	0.913	0.905
	**SD**	0.031	0.033
	**DEV (%)**	33.7	30.6
	**n**	7647	7522
	**Explanatory Variables**	Distance to the coast, SST climatic daily mean, N wind in the sighting location, EKE seasonal Azores	Distance to the coast, chlorophyll week 4, EKE seasonal São Miguel, EKE seasonal Azores
**WEEKLY**	**AUC**	0.918	0.921
	**SD**	0.036	0.018
	**DEV (%)**	38.7	38.2
	**n**	7633	7633
	**Explanatory Variables**	Distance to the coast, SST gradient Azores, SST gradient São Miguel, SST climatic weekly mean, chlorophyll mean Azores, EKE seasonal Azores	Distance to the coast, SST climatic weekly mean, chlorophyll mean Azores, EKE seasonal Azores
**MONTHLY**	**AUC**	0.936	0.88
	**SD**	0.025	0.100
	**DEV (%)**	40.7	40.3
	**n**	7647	7647
	**Explanatory Variables**	Distance to the coast, SST gradient São Miguel, SST gradient Azores, chlorophyll SD Azores, EKE seasonal São Miguel, EKE seasonal Azores	Distance to the coast, SST gradient São Miguel, SST gradient SD São Miguel, Chlorophyll Index 3, chlorophyll month 2 and month 4, EKE seasonal São Miguel, EKE seasonal Azores

AUC: Area Under the Curve of the Receiving Operating Characteristic plot. SD: Standard Deviation of the AUC. DEV(%): Percentage of deviance explained in the model. n: total number of cetacean records (including presence and pseudo-absence) used in the model. SST: Sea Surface Temperature. EKE: Eddy Kinetic Energy.

We found bigger differences among the three high spatial resolution models, the weekly model being the best model of this group. Deviance explained was reasonably good for the three models (daily 30.6%, weekly 38.2% and monthly 40.3%), but when applying the k-fold cross-validation, the monthly model showed a much poorer performance. Its AUC was the smallest of all the implemented models (0.88) and the AUC standard deviation the highest (0.100) (Table 2), which means that for some years this monthly model fits quite well, while for others it fits poorly, leading to substantial differences in AUC among the years considered within the model.

**Fig 9:**
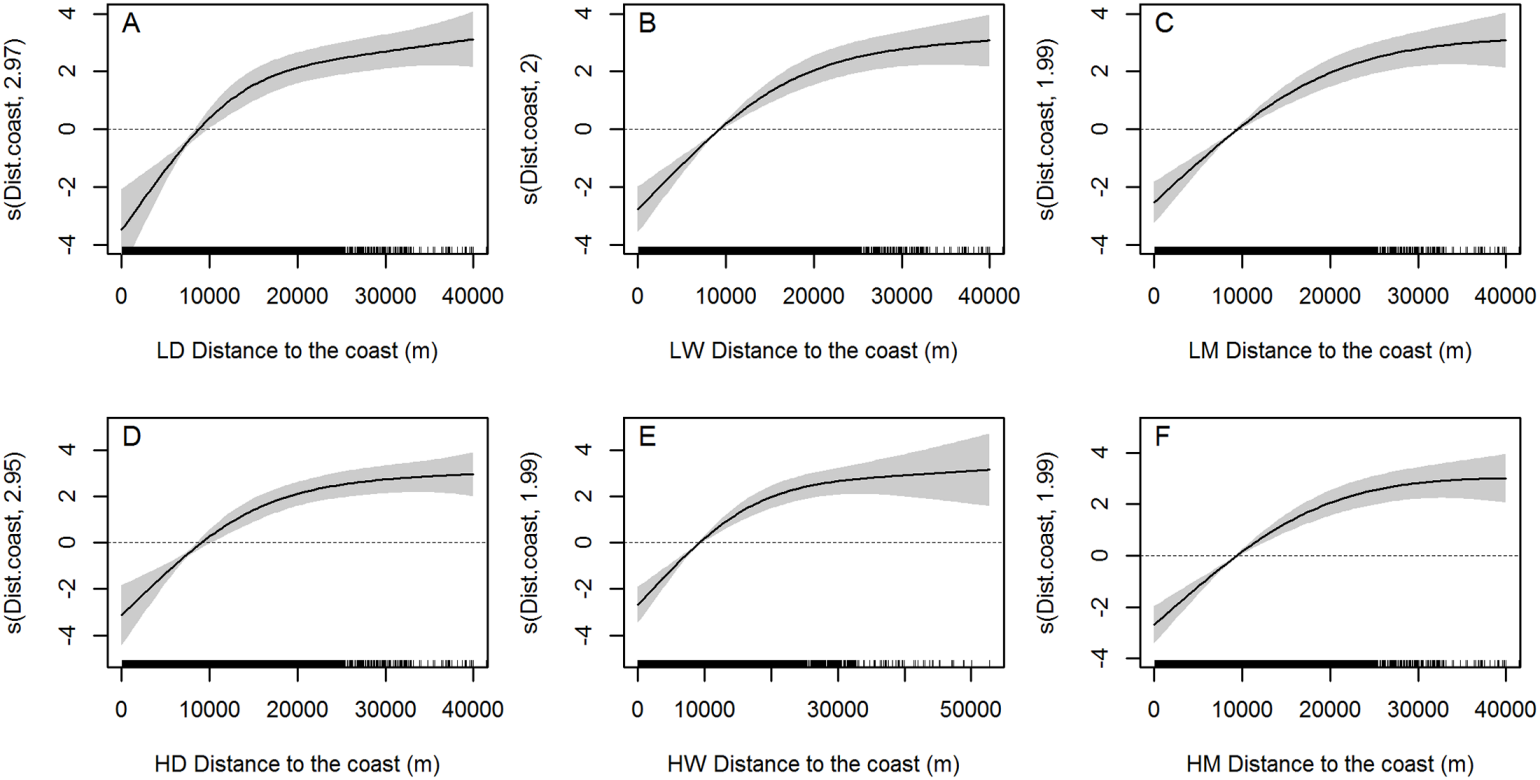
Smoothers showing the effect of distance from the coast on blue whale presence. LD = low spatial daily, LW = low spatial weekly, LM = low spatial monthly, HD = high spatial daily, HW = high spatial weekly and HM = high spatial monthly. Black marks on the x-axis indicate the distribution of our observations.

**Fig 10:**
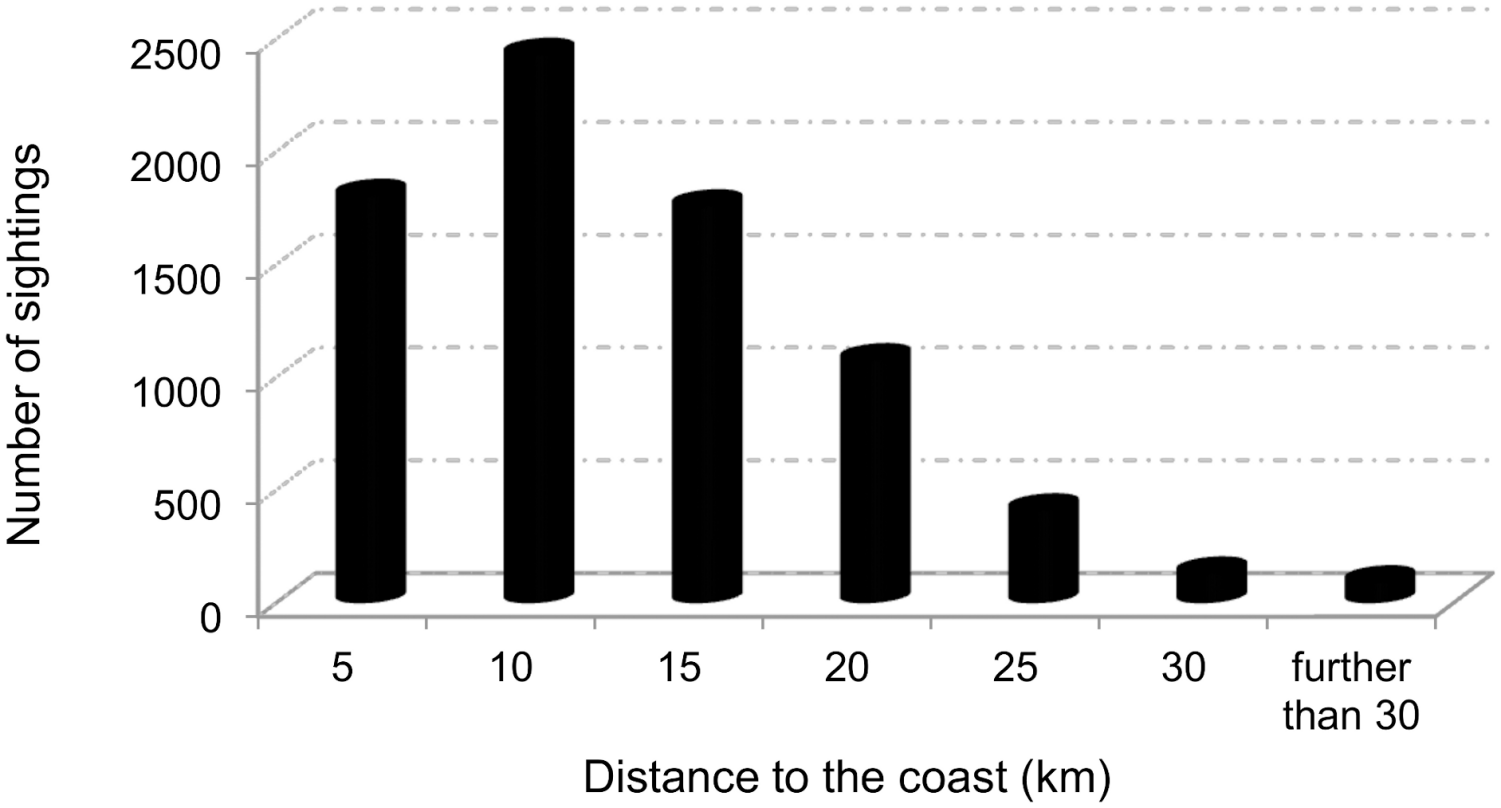
Distance to the coast of the recorded sightings. Most of the sightings were done within 30 km from the coast (98.9%); 77.7% within 15 km from the coast.

**Fig 11:**
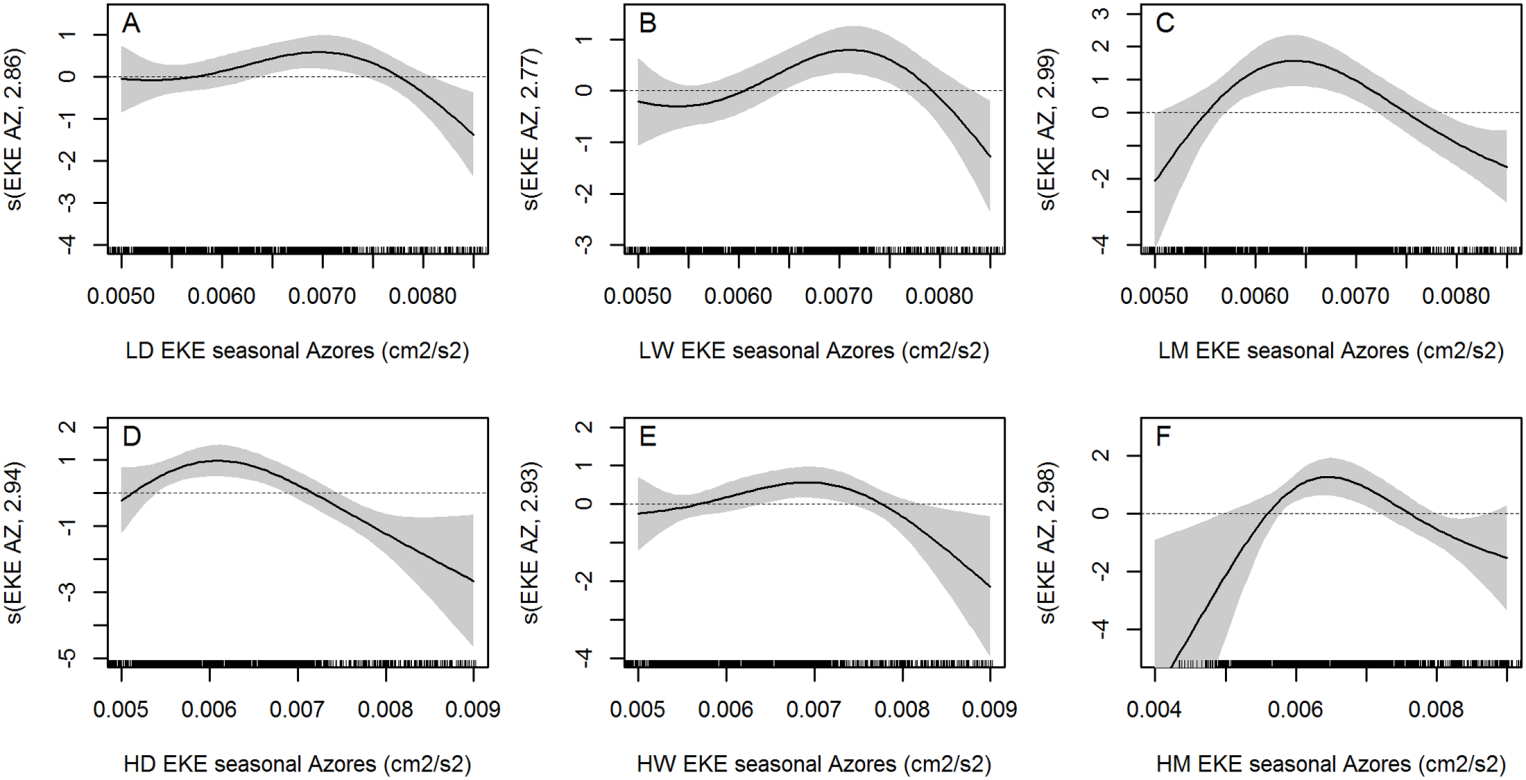
Smoothers showing the effect of seasonal EKE in the Azores archipelago for each of the six final models on blue whale presence. LD = low spatial daily, LW = low spatial weekly, LM = low spatial monthly, HD = high spatial daily, HW = high spatial weekly and HM = high spatial monthly. Black marks on the x-axis indicate the distribution of our observations.

Distance to the coast was selected in all models, both high and low resolution, always with the same shape of the fitted curve, which indicates a preference for waters further than 10km from the coast (Fig 9). To put this in context, observers on land could detect whale blows up to around 30 km offshore but all sightings used in the analysis were from the whale watching boats, which normally travelled up to 30 km from the shore, recording most of the sightings (77.7%) within 15 km from the coast (Fig 10). Therefore, although data might present bias towards shorter distances from the coast due to the initial searching for animals being done from land, blue whales sightings are among those recorded furthest away from land. Seasonal EKE around the archipelago was retained in all the models too. Habitat suitability for blue whales seems to be slightly higher when medium EKE values (60–70 cm^2^s^-2^) are found around the Azores (Fig 11). However, in the vicinity of São Miguel island, the highest available values (>45 cm^2^s^-2^) were preferred.

**Fig 12:**
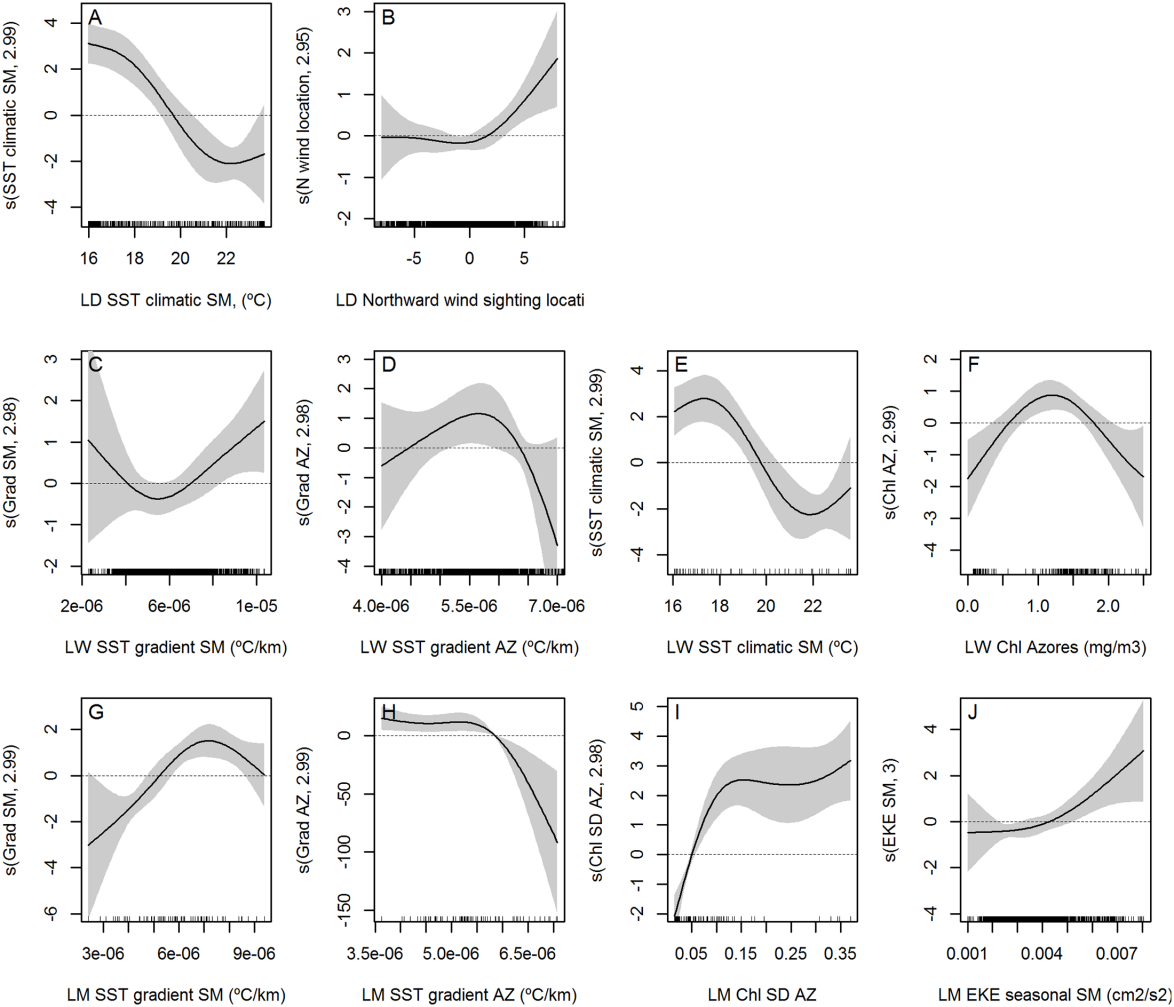
Smoothers of the selected variables in the low spatial resolution final models. All final models include distance to the coast and seasonal EKE for the Azores shown in Figs 9 and 11, apart from the variables here represented. **A-B)** Low spatial daily. **C-E)** Low spatial weekly. **F-H)** Low spatial monthly. Black marks on the x-axis indicate the distribution of our observations.

**Fig 13:**
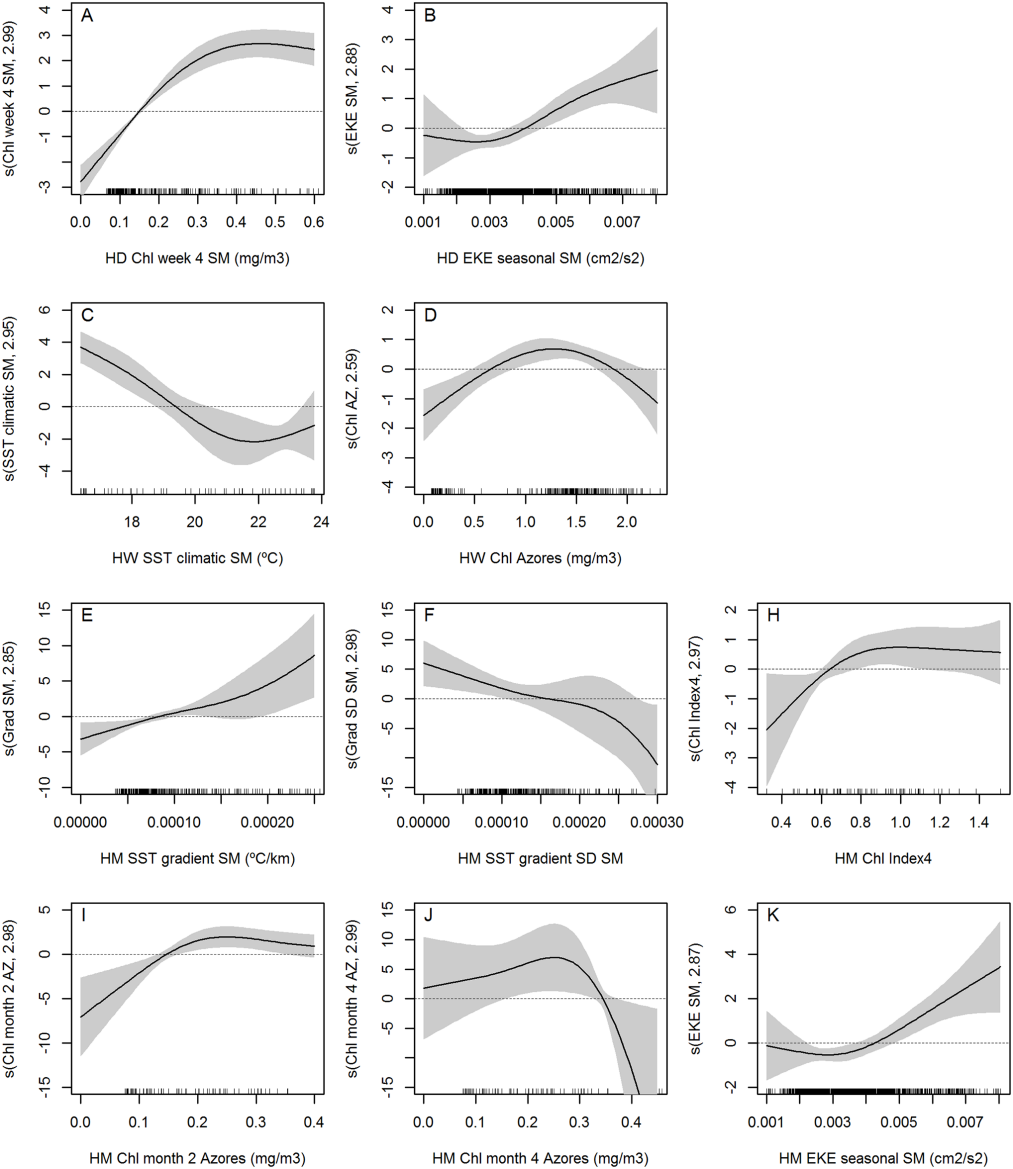
Smoothers of the selected variables in the high spatial resolution final models. All final models include distance to the coast and seasonal EKE for the Azores apart from the variables here represented. **A-B)** High spatial daily. **C-D)** High spatial weekly. **E-I)** High spatial monthly. Black marks on the x-axis indicate the distribution of our observations.

Sea surface temperature gradient was selected for weekly and monthly low resolution models (Fig 12c, 12d, 12g and 12h respectively) and monthly high resolution (Fig 13e). Habitat suitability is increased when low gradient values are found around the archipelago, while stronger gradients are preferred around São Miguel. Climatological SST means were included in three of the final models (low spatial resolution daily and weekly (Fig 12a and 12e), and high spatial resolution weekly (Fig 13c)), with the shapes of the smoothers indicating a preference for waters colder than 19.5°C.

Chlorophyll concentration around the Azores was selected for both weekly models (Figs 12f and 13d). From the range of chlorophyll values available, blue whales prefer medium values around the Azores (1–1.5 mg/m^3^). In the low spatial resolution monthly model, high SD of the chlorophyll concentrations was preferred.

## Discussion

Modelling results can be evaluated from two main perspectives: statistical and ecological performance. The former seeks a robust empirical description of the existence, strength and form of relationships between the response variable (whale presence) and the explanatory variables (environmental conditions); the latter looks for the ecological explanations underlying the observed empirical relationships. Ideally, and as is the case here, statistical and biological performance is linked, because putative explanatory variables are selected on the basis of hypothesised (or at least plausible) relationships between whales and their environment, especially in relation to the distribution of feeding opportunities.

Arguably the biggest challenge relates to the opportunistic nature of the dataset. Initial land search for the animals could bias sightings towards shorter distances from the shore. However, this bias would affect both blue whale sightings and pseudo-absences (sightings of other species), which will tend to counteract the original bias. What is likely is that the number of data points will decline with increasing distance from the shore, resulting in wider confidence limits and a reduced ability to detect preferences. Finally, as our models have a descriptive purpose (and not predictive), we analyze habitat preferences of blue whale in a certain study area, limited in this case by the distance observed from the coast.

The lack of well-quantified effort information directly affects abundance estimations and hampers calculation of encounter rates per unit of area or time. We calculated sightings per number of trips to provide a rough overall abundance index, but did not use this in the models. Pseudo-absences for analyzing habitat preferences can be obtained as random points inside the study area, along the survey route, or using the locations for presence of other species e.g.[67,69,76]. All these methods may present limitations [77], but they allow us to apply modelling techniques (e.g. GAMs) that (evidently) would not be possible without access to absence data. Consequently, we decided to use the sightings of the other species as pseudo-absences of blue whales as done by Esteban *et al*. (2013) [69]. In this way we should minimise biases due to spatial and temporal variation in effort.

Multiple sightings of the same individual are likely to occur. If they happen in a short period of time and within a short distance from each other, over-sampling of the same environmental conditions may occur and true sample sizes (numbers of independent sightings) will be overestimated (i.e. there will be pseudo-replication). For this reason, we deleted all the sightings recorded within 1 hour in near locations, unless different individuals were confirmed with photo-identification. Nevertheless, blue whales can remain around the same area for several days [21] and their behaviour and habitat choice may change during this time. Therefore, one sighting of each whale does not necessarily cover all the habitat conditions it requires.

Abundance of blue whales in the Central and Northeast Atlantic has been estimated at less than 1000 individuals [20], while in the Northwest Atlantic more than 400 blue whales have been identified [78]. In this context, the number of sightings (not to be confused with the number of individuals) used in this study (89) was remarkably high compared with the 17 used by Prieto *et al*. [31] and Tobeña *et al*. [32] or 35 by Visser *et al*. [24] in the same archipelago in previous studies. Furthermore search effort was carried out all year long, with blue whales mostly sighted in spring time, a peak which does not coincide with the period of maximum effort of the year, summer. This implies that this strong seasonality in blue whale sightings around the Azores is not an artefact of the seasonal pattern of search effort.

Another important issue relates to the highly dynamic nature of the marine environment in the study area. The interaction between the dynamic oceanographic conditions and the wide range of sea depths available creates a constantly changing environment that provides a great variety of living conditions throughout the year. As such, the ecological niche of the blue whale encompasses numerous different combinations of conditions, each defining a part of the niche space that is constantly shifting in space and time and which may never co-occur with some other parts of the niche space. In this context of very dynamic domains and highly mobile species which can easily react to those environmental changes, the use of different temporal and spatial scales provides relevant information about the different components of the blue whale niche that otherwise could be overlooked [4,33,35].

In this study low spatial resolution models and the high spatial resolution weekly model performed better than the high spatial resolution daily and monthly ones. In general, high spatial resolution models were less consistent, and indeed following an objective model selection process became more difficult for the high spatial resolution data sets, due to several different models having similar goodness of fit. Although this could indicate that the model is failing to capture the underlying ecological processes, we suspect that an important issue is the quality of the remote-sensing data, which presented many missing values caused by the frequent cloud coverage in the Azores. This issue implied a considerable decrease of the number of sightings for which environmental values were available (95.35% of the sightings lost for the high resolution daily model). This argument is supported by some other recent studies in the Azores [31,32], which avoided the use of high resolution satellite-derived variables due to these practical limitations. Nevertheless, we decided to consider two different spatial resolutions for sea surface temperature, which we expected to be an important variable affecting blue whale distribution. High spatial resolution data allow us to recognize meso- and submesoscale oceanographic features (with dimensions of dozens to hundreds of km) known to affect our study area [53,55]. With coarser scales, they could be overlooked. Those features sometimes last just for a few days (or even hours) but, even if ephemeral, they can have a profound effect on the ocean productivity, and thus on species distribution. For this reason we argue in favour of trying daily variables, as blue whales are highly mobile species able to easily react to short-term changes in the environment.

### Ecological meaning

Only two of the explanatory variables were included in all the final models: distance to the coast (Fig 8) and seasonal EKE for the Azores (Fig 10). In all models blue whales show a preference for waters further than 10 km from the coast, which are usually deep waters. EKE represents a good proxy for mesoscale eddy activity [55], so the preference for medium to high EKE values around the archipelago leads us to think about the power of eddies to aggregate plankton and potentially enhance food availability.

In weekly models, medium values of chlorophyll in the archipelago were preferred. High chlorophyll concentrations are generally related with the phytoplankton bloom and, thus, the first link of the trophic chain. Blue whales feed almost exclusively on krill *(Euphausiacea)* [10,79], and this zooplankton family, the distribution of which is directly affected by the oceanographic dynamism of the area, needs some time to develop after the bloom leading to a noticeable reduction of the chlorophyll concentration in the area at the time the whales are present. This would explain the apparent preference of the whales for locations with medium values of chlorophyll concentration instead of higher ones. Furthermore, blue whales are known to feed not only on the surface, but also at depths of up to almost 300 m [80]. Chlorophyll distribution in the water column depends on local oceanography and its interaction with the bathymetry, and the concentration at depth will not always agree with its surface signature [81,82].

In the low spatial resolution monthly model, there was a preference for high values of chlorophyll SD, meaning areas where chlorophyll is patchily distributed. Supporting this idea, the preference for quite strong SST gradients around São Miguel (low spatial resolution monthly model), together with the high EKE around the island (high spatial resolution monthly model), suggests that dynamic environments, thus with the presence of oceanographic features like eddies (normally linked with EKE), filaments or fronts (normally linked with high SST gradients), could result in more suitable habitats for blue whales. Those features can be very relevant when or where oligotrophic conditions are present, implying convergence or aggregation zones which can increase food availability [4,83].

We agree with Fernández [34], who suggested the use of weekly or daily scales in order to acquire useful results for highly mobile species in dynamic environments. However, in our study the monthly low resolution model also seems to perform well for blue whales. This could be caused by the location of our study area, in the eastern group of the Azores, which is particularly influenced by a westward flow of eddies derived from the Azores Current with a life span of more than six months [55] and a noteworthy aggregation power [53,84]. Therefore, a great part of this variability, and hence, of its effects on whale distribution, may be captured by monthly variables for this region.

### Temporal distribution

We confirmed the presence of blue whales every year around the Azores in spring time, agreeing with previous studies in the area [21,24,31]. During spring, it is known that at least some blue whales interrupt their migration towards the North Atlantic feeding grounds to forage around the archipelago before continuing their journey [21]. Our results support this hypothesis, highlighting, in all the final habitat preference models the importance of dynamic variables responsible for the availability and distribution of whales prey. These habitat preferences cannot be therefore generalized to those favoured by the species while travelling [54].

After summer, blue whales are supposed to migrate to their low latitude breeding grounds, but as sightings in autumn are extremely rare in the Azores (only 1 sighting in September 2012) they probably follow different routes, as suggested by the recent findings off NW Iberia, where several blue whales have been sighted in September and October 2017 [85].

## Conclusions

Our results suggest that multiscale studies are very useful in species distribution modelling for highly mobile species which exploit dynamic habitats. With the use of several temporal and spatial scales, we increase chances of capturing relevant oceanographic structures or environmental changes likely to affect whale distribution, both persistent and larger events (e.g. spring bloom) and more localized and short-term ones (e.g. mesoscale eddies or local upwelling). We compared daily, weekly and monthly resolutions with two different spatial scales. Weekly composites of satellite-derived oceanographic variables turned out to be a valid option for both spatial scales. Daily data were useful as well for both spatial scales, as final models didn’t retain chlorophyll or SST variables, the ones that considerably reduce sample size due to the inherent data gaps in the environmental products.

Blue whales have a well-defined ecological niche around the Azores. They prefer deep waters further from the coast (>10 km) within cold temperatures (typical in spring time). The importance of dynamic areas, which can enhance productivity and promote convergence zones, is revealed by medium to high EKE values included in all the final models. All the models obtained within this study show a good statistical performance: reasonably high deviance explained (between 31.0 and 41.6%) and reasonably good AUC (from 0.822 ± 0.024 to 0.931 ± 0.024); and generally made sense ecologically.
